# Aberrant expression of NKL homeobox gene HLX in Hodgkin lymphoma

**DOI:** 10.18632/oncotarget.24512

**Published:** 2018-02-16

**Authors:** Stefan Nagel, Claudia Pommerenke, Corinna Meyer, Maren Kaufmann, Roderick A.F. MacLeod, Hans G. Drexler

**Affiliations:** ^1^ Department of Human and Animal Cell Lines, Leibniz-Institute DSMZ-German Collection of Microorganisms and Cell Cultures, Braunschweig, Germany

**Keywords:** homeobox, NKL-code, T-ALL

## Abstract

NKL homeobox genes are basic regulators of cell and tissue differentiation, many acting as oncogenes in T-cell leukemia. Recently, we described an hematopoietic NKL-code comprising six particular NKL homeobox genes expressed in hematopoietic stem cells and lymphoid progenitors, unmasking their physiological roles in the development of these cell types. Hodgkin lymphoma (HL) is a B-cell malignancy showing aberrant activity of several developmental genes resulting in disturbed B-cell differentiation. To examine potential concordances in abnormal lymphoid differentiation of T- and B-cell malignancies we analyzed the expression of the hematopoietic NKL-code associated genes in HL, comprising HHEX, HLX, MSX1, NKX2-3, NKX3-1 and NKX6-3. Our approach revealed aberrant HLX activity in 8 % of classical HL patients and additionally in HL cell line L-540. Accordingly, to identify upstream regulators and downstream target genes of HLX we used L-540 cells as a model and performed chromosome and genome analyses, comparative expression profiling and functional assays via knockdown and overexpression experiments therein. These investigations excluded chromosomal rearrangements of the HLX locus at 1q41 and demonstrated that STAT3 operated directly as transcriptional activator of the HLX gene. Moreover, subcellular analyses showed highly enriched STAT3 protein in the nucleus of L-540 cells which underwent cytoplasmic translocation by repressing deacetylation. Finally, HLX inhibited transcription of B-cell differentiation factors MSX1, BCL11A and SPIB and of pro-apoptotic factor BCL2L11/BIM, thereby suppressing Etoposide-induced cell death. Collectively, we propose that aberrantly expressed NKL homeobox gene HLX is part of a pathological gene network in HL, driving deregulated B-cell differentiation and survival.

## INTRODUCTION

Human blood cells originate in the bone marrow where hematopoietic stem cells (HSC) generate ancestors of both the myeloid and lymphoid lineages. The common lymphoid progenitors (CLPs) differentiate into B-cells, T-cells or natural killer (NK)-cells. NK-cells usually complete their development in the bone marrow, early T-cell progenitors migrate to the thymus for subsequent differentiation while naïve B-cells terminate their maturation in lymph nodes. These lymphocyte-specific localizations indicate that external signals have major impacts on lineage-differentiation via particular signalling pathways. The developmental processes of lymphopoiesis are mainly regulated at the transcriptional level [1, 2]. Accordingly, lymphocyte differentiation depends on activities of lineage-specific transcription factors (TFs) like PAX5 and BCL11A for B-cells, GATA3 and BCL11B for T-cells, and ID2 and NFIL3 for NK-cells [1, 3, 4]. Of note, some TF families are of conspicuous and widespread importance for lymphopoiesis like helix-loop-helix (HLH) factors [5, 6]. Thus, deregulated activity of HLH proteins plays a role in several lymphoid malignancies including Hodgkin lymphoma (HL) [5–7].

In HL infiltrated lymph nodes contain just a small number of the malignant Hodgkin/Reed-Sternberg (HRS) cells and many bystander cells, including activated lymphocytes, plasma cells and granulocytes [8]. This situation reflects aberrant expression of several signalling molecules like interleukins and other growth factors together with their receptors, resulting in constitutive activation of the associated pathway mediators including JAK-STAT [9–11]. Additionally, aberrant activities of NFkB TFs promote survival of the HRS cells. Multiple mechanisms have been described which contribute to their activation in HL, including amplification of REL and mutation of TNFAIP3/A20 [12, 13]. According to clinical and histopathological features HL is classified in two distinct entities, comprising classical HL (cHL) and nodular lymphocyte predominant HL (NLPHL) [14].

From analysis of gene expression profiles of cell lines and microdissected primary HRS cells compromised B-cell development has been highlighted as a major aspect of the pathogenesis in HL [15–17]. Main TFs important for B-cell development are absent or inactivated, resulting in B-cells with incomplete phenotypes [7]. Aberrantly downregulated B-cell TFs include PAX5, BOB1/OBF1, OCT2 and EBF1 [17–19]. Suppression of PAX5, BOB1 and OCT2 is responsible for the loss of immunoglobulin expression accompanying blocked B-cell development [20]. Additional features of disturbed B-cell differentiation in HL include repression of the activity of basic HLH protein TCF3/E2A by overexpressed ID2 and ABF1 and ectopic activation of T-cell specific TF GATA3 [7, 21]. However, reactivation of the fundamental TF PAX5 is alone insufficient to reinstate the B-cell program in HL, indicating that multiple factors are involved in coordinating B-cell differentiation [22].

Malignant cells of T-cell acute lymphoblastic leukemia (T-ALL) are developmentally arrested thymocytes expressing stage-specific genes and particular oncogenes [23]. Homeobox genes TLX1, TLX3, NKX2-1 and NKX2-5 encode oncogenic TFs in T-ALL which are physiologically silenced during hematopoiesis, but undergo ectopic activation in transformed thymocytes [24, 25]. NKX3-1 and MSX1 are physiologically expressed in hematopoietic stem and progenitor cells and remain aberrantly active in T-ALL subsets [26]. The aforementioned genes belong to the 48 member strong NKL subclass of homeobox genes which numbers to date more than 20 aberrantly expressed genes in T-ALL [25, 27, 28]. Thus, NKL homeobox members represent the largest single class of oncogenes in T-ALL.

NKL homeobox genes regulate fundamental processes in both embryonal development and differentiation in the adult. Some represent master genes for specific cell types/organs like NKX2-3 (spleen), NKX2-5 (heart), or NKX3-1 (prostate), others operate a code which regulates the development of complex structures or tissues [29–32]. Accordingly, we have coined the term NKL-code which describes the physiological expression pattern of particular NKL homeobox genes in early hematopoiesis and lymphopoiesis [25]. Thus, due to their basic impact, aberrant activity patterns of NKL homeobox genes potentially contribute to leukemogenesis/lymphomagenesis by deregulating developmental processes.

Here, we analyzed hematopoietic NKL-code associated homeobox genes in HL and identified aberrantly activated HLX in a subset of patients. We used a HLX expressing HL cell line as a model and identified upstream regulators and downstream targets. Our data assemble a pathological gene regulatory network surrounding HLX and highlight the NKL homeobox gene subclass in this type of B-cell lymphoma.

## RESULTS

### Aberrant expression of HLX in HL

**Figure 1:**
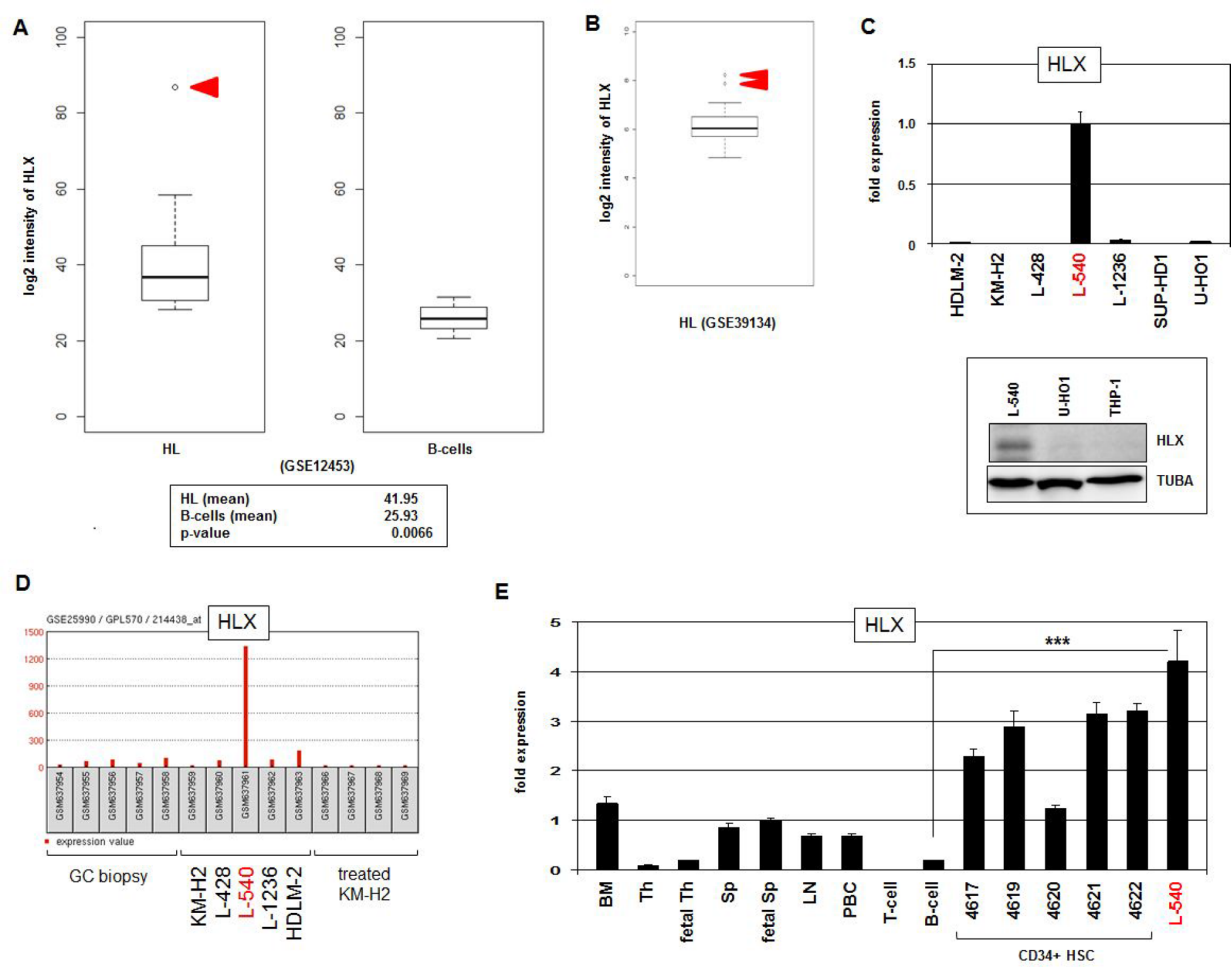
HLX expression in HL (**A**) HL patient samples (*N* = 17) in comparison to normal B-cell controls (*N* = 25) from public gene expression profiling dataset GSE12453 were analyzed for HLX expression levels. The data demonstrate significantly higher HLX expression in HL and 1/12 cHL patient sample with conspicuously enhanced gene activity (red arrow head). (**B**) Overexpression of HLX was detected in 2/29 (7%) of cHL patients (red arrow heads) in dataset GSE39134. (**C**) HLX transcription was quantified by RQ-PCR in seven HL cell lines, demonstrating a strong signal in L-540 cells (above). HLX protein was verified in L-540 cells by Western blot. Tubulin served as control (below). (**D**) HLX gene activity was analyzed in public dataset GSE25990, demonstrating an outstanding signal in HL cell line L-540 while control HL cell lines and primary GC biopsy samples indicated absent or weak transcription. (**E**) RQ-PCR analysis of HLX was performed for HL cell line L-540 in comparison to primary hematopoietic cell/tissue samples, comprising bone marrow (BM), thymus (Th), fetal thymus, spleen (Sp), fetal spleen, lymph node (LN), peripheral mononuclear blood cells (PBC), T-cells, B-cells, and CD34-positive HSCs. The HLX expression level of fetal spleen was set to 1.

To identify aberrant NKL homeobox gene activities in HL patients we analyzed the expression levels of those six subclass members which constitute the hematopoietic NKL-code, comprising HHEX, HLX, MSX1, NKX2-3, NKX3-1 and NKX6-3 [25]. We scrutinized public gene expression dataset GSE12453 which contains in addition to 17 microdissected HL cases (12 cHL and 5 NLPHL), 4 cases of T-cell rich B-cell lymphoma, 5 follicular lymphoma, 5 Burkitt lymphoma (BL), 11 diffuse large B-cell lymphoma (DLBCL), and 25 normal B-cell samples representing different developmental stages obtained from peripheral blood and tonsils (Supplementary Figure 1). The data indicated that HHEX expression levels were reduced in all types of B-cell lymphoma, excluding oncogenic activation. In contrast, overexpression of HLX was detected in at least one cHL patient and one DLBCL patient. Another case of DLBCL showed NKX2-3 overexpression and one BL patient overexpressed NKX6-3. NKX3-1 expression values, however, suggested no convincing pattern of deregulation. The data for MSX1 demonstrated varying expression levels indicating rather aberrantly suppressed gene activity. Thus, particular NKL homeobox genes showed aberrant activities in B-cell malignancies - in HL we clearly identified overexpression of HLX in 1/12 (8 %) cHL patients (Figure 1A). Statistical analysis of the HLX expression data demonstrated significantly elevated activity in HL as compared to normal B-cells (*p* = 0.0066). Moreover, analysis of dataset GSE39134 which contains 29 cHL cases showed HLX overexpression in 2/29 (7%) patients (Figure 1B), supporting our findings and the clinical relevance of this deregulated homeobox gene in the main entity of HL.

Next we quantified by RQ-PCR HLX expression in seven HL cell lines (HDLM-2, KM-H2, L-428, L-540, L-1236, SUP-HD1, U-HO1), finding conspicuously high transcript levels in L-540. HLX expression in this cell line was also detectable at the protein level by Western blot (Figure 1C), supporting functional potential. Public dataset GSE25990 comprises in addition to samples from HL cell lines biopsies from primary normal germinal centres (GC). Analysis of these data confirmed aberrant activation of HLX in L-540 contrasting with cell line controls and primary samples alike (Figure 1D). RQ-PCR analysis of HLX in various primary hematological cell types/tissues showed likewise high expression levels in HSCs in comparison to L-540, while expression in mature B-cells was minimal (Figure 1E). Significant HLX expression was also detected in the bone marrow, confirming its previously described physiological activity in the stem cell compartment [25, 33]. Taken together, in a subset of cHL patients and HL cell line L-540 we identified aberrant activity of NKL homeobox gene HLX indicating its pathological relevance in this malignancy. In the following we used L-540 cells to reveal upstream and downstream factors of HLX in HL.

### STAT3 activates HLX expression

**Figure 2:**
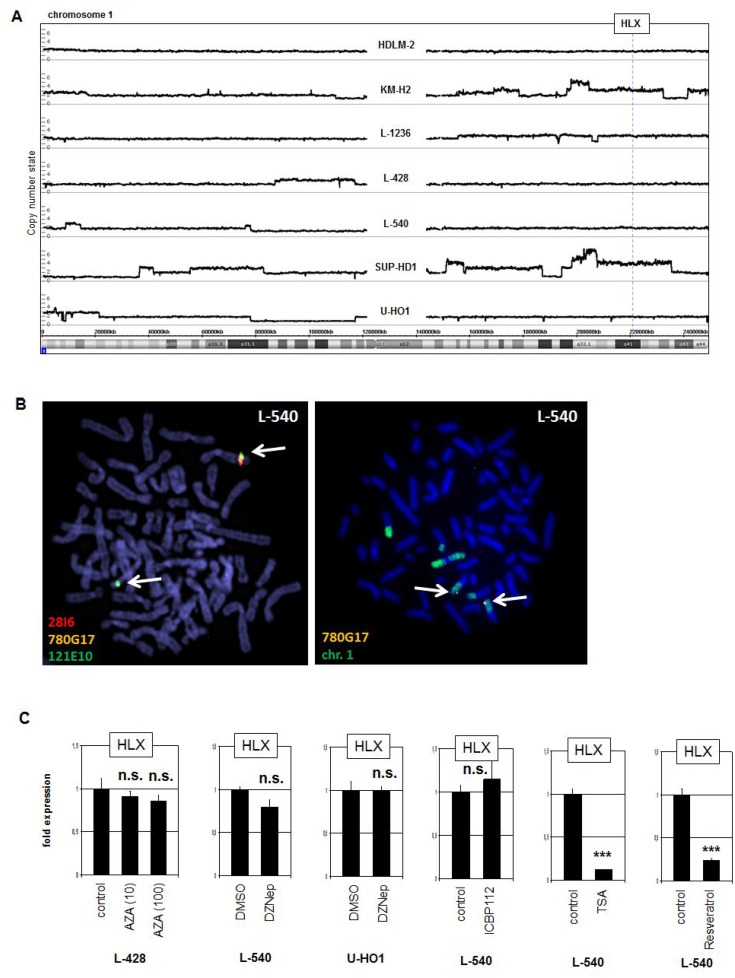
Genomic, chromosomal and chromatin analyses (**A**) Genomic profiling was performed for seven HL cell lines. The results for chromosome 1 demonstrated absence of copy number alterations in L-540 at the locus of HLX (1q41). (**B**) Fluorescence *in-situ* hybridization (FISH) in L-540 demonstrated wild type configurations at the locus of HLX although chromosome 1 showed some structural abnormalities. We used two flanking and one spanning probe for HLX (left), and the spanning probe in combination with a painting probe for chromosome 1 (right). The clone names and the colors of the used probes are indicated. The detected loci of HLX are indicated by arrows. (**C**) RQ-PCR analysis of HLX was performed for HL cell line L-540 after treatment with various inhibitors.

In T-ALL NKL homeo-oncogenes are frequently activated via chromosomal aberrations [34]. To examine if HLX is similarly activated in L-540 we performed genomic profiling (Figure 2A) and fluorescence *in-situ* hybridization (Figure 2B). However, the HLX locus at 1q41 showed absence of copy number gain and of chromosomal translocation in L-540 cells, excluding activation via genomic/chromosomal abnormalities. Of note, copy number gain at 1q22-42 in SUP-HD1 did not result in aberrant HLX expression, suggesting that specific TFs or chromatin configurations are necessary for activation.

To analyze potential impacts by DNA- and chromatin-modifying factors we treated HL cell lines with drugs specifically inhibiting DNA-methylation (AZA), histone methyltransferase EZH2 (DZNep), histone acetyltransferase EP300 (ICBP112) and histone deacetylases (TSA and Resveratrol). Excepting TSA and Resveratrol these drugs used did not significantly change the expression levels of HLX (Figure 2C). But the latter two drugs strongly suppressed HLX transcription excluding deacetylation of histones as direct regulatory mechanism and indicative that reactivation of a suppressive TF or acetylation of a non-histone TF strongly inhibits its HLX-regulating potential (see below).

To identify potential upstream factors of HLX we conducted expression profiling of L-540 in comparison to HL cell lines KM-H2, L-428, L-1236, SUP-HD1 and U-HO1. Then we performed gene-annotation enrichment analysis of the top 1000 differentially expressed genes. The results obtained indicated activation of JAK-STAT signalling with high statistical significance in addition to cytokine-driven pathways (Supplementary Figure 2). Moreover, detailed analysis of overexpressed genes in L-540 highlighted adrenoreceptor-signalling in addition to several ligands/receptors known to activate JAK-STAT, namely IGF1, IFNG, IL2, IL6, IL7, IL12 and IL22 (Supplementary Table 1). However, treatments of L-540 cells with cognate ligands, inhibitory antibodies or drugs failed to uncover any role in HLX activation (Supplementary Figure 3).

**Figure 3:**
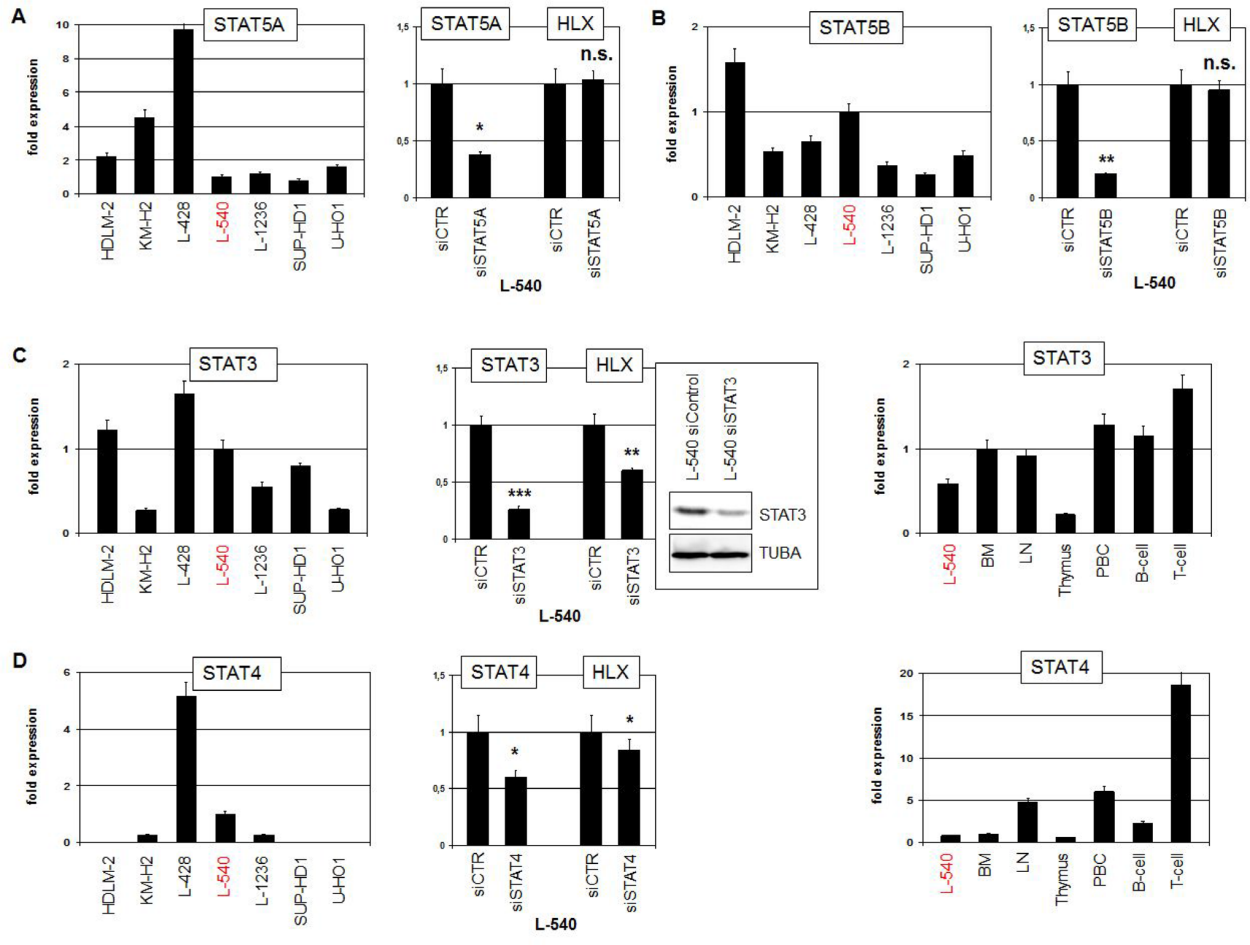
STAT-factors in HL (**A**) RQ-PCR analysis of STAT5A expression in HL cell lines (left). RQ-PCR analysis of STAT5A and HLX in L-540 cells treated for siRNA-mediated knockdown of STAT5A (right). (**B**) RQ-PCR analysis of STAT5B expression in HL cell lines (left). RQ-PCR analysis of STAT5B and HLX in L-540 cells treated for siRNA-mediated knockdown of STAT5B (right). (**C**) RQ-PCR analysis of STAT3 expression in HL cell lines (left). RQ-PCR and Western blot analysis of STAT3 and HLX in L-540 cells treated for siRNA-mediated knockdown of STAT3 (middle). RQ-PCR analysis of STAT3 in L-540 and primary hematopoietic cells/tissues, including bone marrow (BM), lymph node (LN), thymus, PBC, B-cells and T-cells (right). (**D**) RQ-PCR analysis of STAT4 expression in HL cell lines (left). RQ-PCR analysis of STAT4 and HLX in L-540 cells treated for siRNA-mediated knockdown of STAT4 (middle). RQ-PCR analysis of STAT4 expression in L-540 and primary hematopoietic cells/tissues (right).

To examine directly the impact of STAT factors we quantified expression levels and performed siRNA-mediated knockdown of STAT5A, STAT5B, STAT3 and STAT4 (Figure 3). STAT5A expression was detected in all analyzed HL cell lines at varying levels. However, siRNA-mediated knockdown of STAT5A did not change the expression of HLX in L-540, excluding an activating role for this factor (Figure 3A). Expression levels of STAT5B maintained a similar range in HL cell lines. But again, STAT5B knockdown experiments excluded any regulatory impact on HLX expression (Figure 3B). HDLM-2, L-428 and L-540 expressed elevated STAT3 levels while in the remaining HL cell lines lower levels were detected. STAT3 knockdown resulted in decreased HLX transcript levels, demonstrating that STAT3 activated the expression of HLX (Figure 3C). Finally, STAT4 expression was conspicuously high in L-428, detectable in L-540, L-1236 and KM-H2 and absent in HDLM-2, SUP-HD1 and U-HO1. STAT4 knockdown resulted in slightly decreased HLX transcript levels, demonstrating that STAT4 activated HLX as well, albeit moderately (Figure 3D). Expression analysis of STAT3 and STAT4 in primary hematological samples showed a more or less uniform activity for STAT3, while STAT4 exhibited enhanced activity in T-cells (Figure 3C, 3D). These data may indicate that in B-cells STAT3 plays a more prominent role than STAT4. Of note, both STAT3 and STAT4 showed overexpression in HL patient samples as compared to normal B-cells (Supplementary Figures 4, 5), reflecting their expression pattern in HL cell lines and supporting a pathological role for these TFs [11, 35].

**Figure 4:**
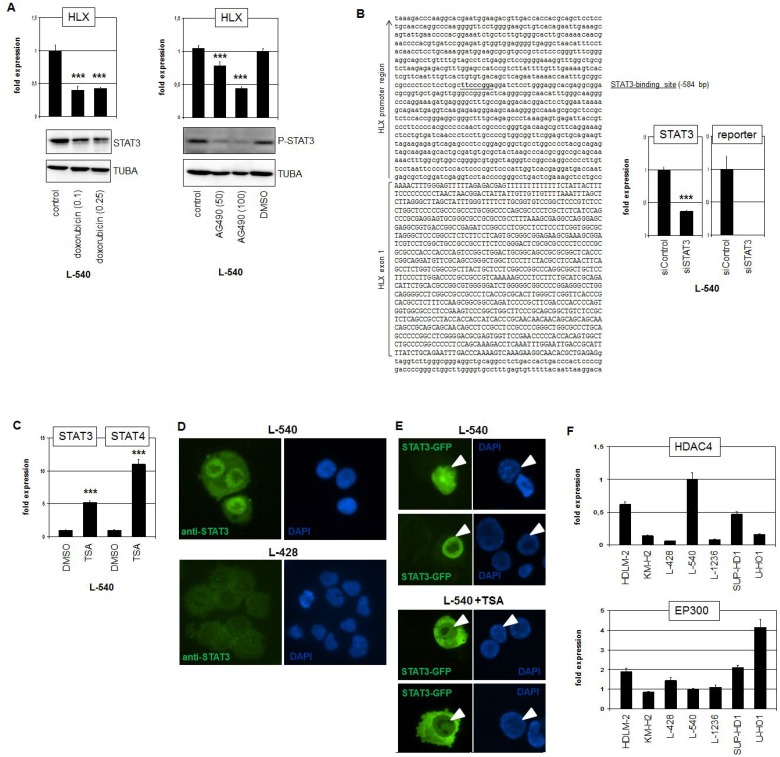
STAT3 activates HLX in HL (**A**) RQ-PCR analysis of HLX in L-540 cells after treatment with 0.1 or 0.25 μM Doxorubicin (left) and 50 or 100 μM AG490 (right). Western blot analyses demonstrate the treatment effects on STAT3 protein level (left) and on phosphorylation of STAT3 (right). Tubulin served as control. (**B**) Reporter-gene assay for the regulation of HLX expression by STAT3. The promoter region of HLX contains a potential STAT3-site (underlined) at −584 bp (left). RQ-PCR analysis of L-540 cells transfected with reporter-gene construct and siRNA (right). The expression level of STAT3 was reduced via siRNA-mediated knockdown and the activity of the reporter-gene decreased concurrently, demonstrating direct activation of HLX by STAT3. (**C**) RQ-PCR analysis of STAT3 and STAT4 in L-540 cells after treatment with deacetylase-inhibitor TSA. (**D**) Immunofluorescence analysis of L-540 and L-428 cells using STAT3-antibody (green) and DAPI as nuclear counterstain (blue). Please note the strong nuclear STAT3-signal in L-540. (**E**) Fluorescence analysis of L-540 cells transfected with GFP-tagged STAT3. Treatment of these cells with TSA resulted in translocation of STAT3-GFP into the cytoplasm (below), while the controls show nuclear localization of STAT3-GFP (above). Nuclei of STAT3-GFP positive cells are indicated by arrowheads. (**F**) RQ-PCR analysis of HDAC4 (above) and EP300 (below) in HL cell lines, demonstrating elevated and reduced expression levels, respectively, in L-540 cells.

To confirm the regulatory impact of STAT3 in HLX expression we treated L-540 cells with two different STAT3-inhibitors. Doxorubicin mediates degradation of the STAT3 protein while AG490 inhibits its activating phosphorylation. Treatments of L-540 cells with either drug showed the described effects on STAT3 and resulted in reduced expression levels of HLX (Figure 4A), confirming the knockdown data. ENCODE ChIP-seq data of EBV-transfected B-cells (GM12878) show STAT3 binding in the promoter region of HLX (Supplementary Figure 6) [36]. Accordingly, sequence analysis of this region revealed a potential STAT3 binding site at position −584 bp. This finding prompted performance of a reporter gene assay using a genomic fragment containing this site as driver. The combination of the promoter-reporter-construct with siRNA directed against STAT3 indicated a direct activation of HLX by STAT3 via this site (Figure 4B).

### Regulation of STAT3 activity by subcellular localization

As described above, treatment of L-540 with deacetylase-inhibitor TSA (or Resveratrol) resulted in decreased HLX expression levels (Figure 2C). Surprisingly, TSA treatment even increased the expression levels of STAT3 and STAT4 (Figure 4C), giving no explanation for its inhibitory effect on HLX. However, immunofluorescence analysis of STAT3 indicated that L-540 cells expressed higher protein levels with concentrated nuclear presence as compared to L-428 (Figure 4D). To investigate if acetylation of STAT3 protein impacts its subcellular localization we transfected an expression-construct encoding GFP-tagged STAT3 into L-540 cells which were then treated with TSA (Figure 4E). While the STAT3 fusion-protein localized to the nucleus of transfected control cells it underwent cytoplasmic localization after treatment with TSA. Thus, the nuclear localization of STAT3 protein is performed via deacetylation. According to prominent nuclear localization of STAT3 in cells with enhanced deacetylase-activity, L-540 expressed high levels of deacetylase HDAC4 (Supplementary Table 1) and low levels of acetyltransferase EP300 (Figure 4F), which may support STAT3 deacetylation. Similarly, expression profiling data of primary HL and control B-cells showed significantly higher HDAC4 and lower EP300 levels in HL patient samples as found in HL cell lines underlining the clinical relevance of these genes (Supplementary Figure 4, 5). Of note, SIRT1 (inhibited by Resveratrol) eschewed elevated in favor of reduced expression levels in HL patient samples (Supplementary Figure 4), suggesting that this gene is not involved in STAT3 deacetylation in primary HL cells.

Finally, STAT3 protein position K685 has been identified as a critical acetylation site which controls subcellular localization [37]. However, sequence analysis of L-540 revealed only wildtype sequence at this position (data not shown), which might have contributed to a loss of acetylation-mediated translocation into the cytoplasm. Moreover, sequence data from the public COSMIC database (Wellcome Trust Sanger Institute, UK) failed to show any mutations in the complete STAT3 gene of L-540. Collectively, our results showed that activated/phosphorylated and nuclear/deacetylated STAT3 directly regulates HLX transcription. However, mechanisms mediating elevated STAT3 expression levels and protein activation in L-540 remained unclear.

### HLX suppresses B-cell differentiation and apoptosis in HL

To identify downstream targets of HLX we reanalyzed our expression profiling data of L-540 in comparison to controls. Our previous study about NKL homeobox genes in hematopoiesis indicated that HLX operates in progenitor cells as a repressor [25]. To follow this assumption we screened the top 1000 differentially downregulated genes and selected promising candidates for detailed analyses including MSX1, BCL11A, SPIB, and BCL2L11/BIM (Supplementary Table 2).

**Figure 5:**
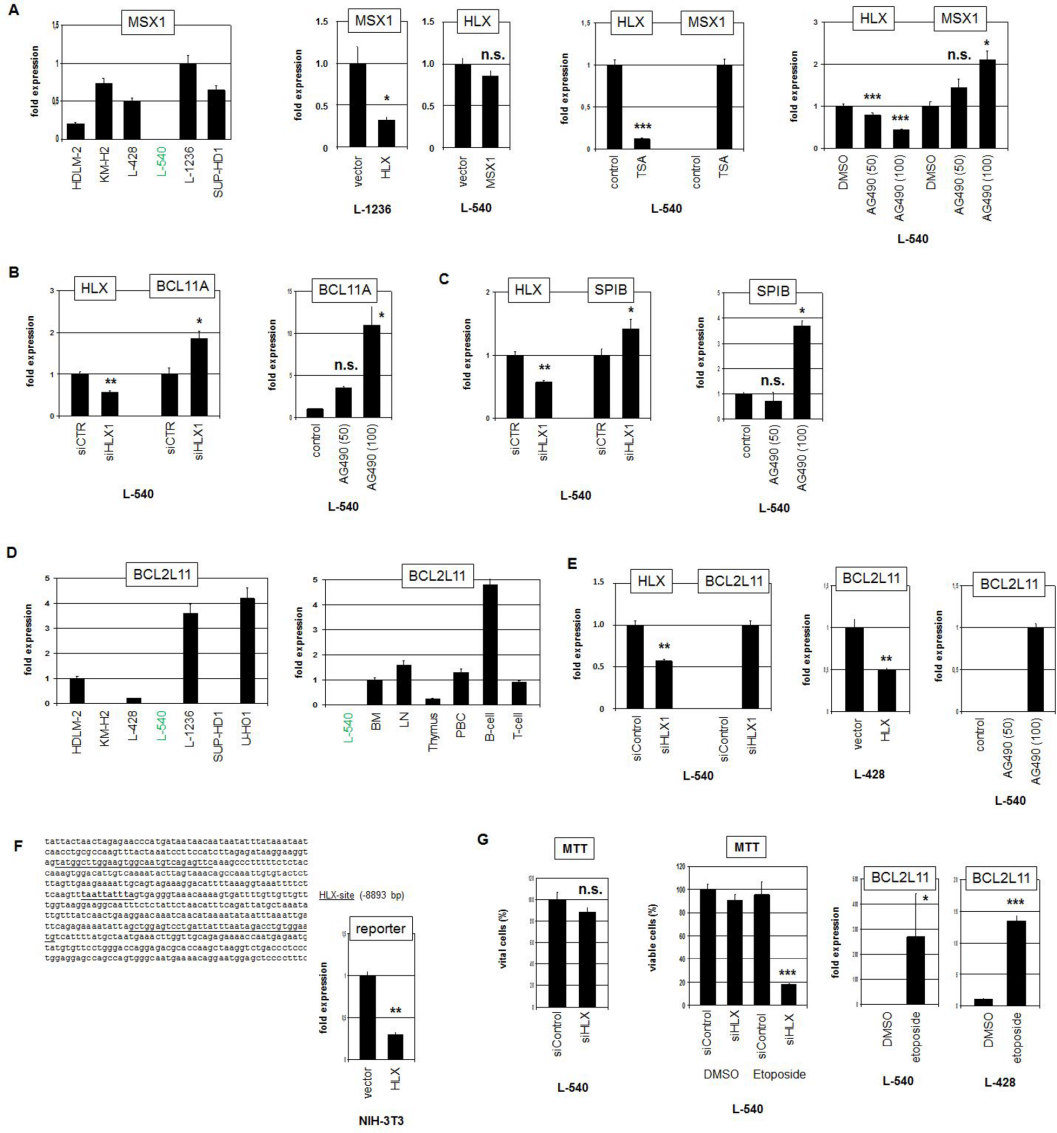
Target genes of HLX in HL (**A**) RQ-PCR analysis of MSX1 in HL cell lines, demonstrating low/absent expression level in L-540 (left). Forced expression of HLX in L-1236 resulted in reduced transcript levels of MSX1, while forced expression of MSX1 in L-540 showed no change of HLX expression level (middle left). RQ-PCR analysis of L-540 cells treated with TSA resulted in reduced expression of HLX and concurrently increased expression of MSX1 (middle right). RQ-PCR analysis of L-540 cells treated with STAT3-inhibitor AG490 resulted in reduced expression of HLX and concurrently increased expression of MSX1 (right). These data indicated that HLX suppressed the expression of MSX1. (**B**) RQ-PCR analysis of HLX and BCL11A in L-540 cells after treatment for siRNA-mediated knockdown of HLX (left). RQ-PCR analysis of L-540 cells treated with STAT3-inhibitor AG490 resulted in increased expression of BCL11A (right). These data indicated that HLX suppressed the expression of BCL11A. (**C**) RQ-PCR analysis of HLX and SPIB in L-540 cells after treatment for siRNA-mediated knockdown of HLX (left). RQ-PCR analysis of L-540 cells treated with STAT3-inhibitor AG490 resulted in increased expression of SPIB (right). These data indicated that HLX suppressed the expression of SPIB. (**D**) RQ-PCR analysis of BCL2L11 in HL cell lines demonstrating low/absent expression levels in L-540 cells (left). RQ-PCR analysis of BCL2L11 in L-540 and primary hematopoietic cells/tissues, including bone marrow (BM), lymph node (LN), thymus, PBC, B-cells and T-cells (right). (**E**) RQ-PCR analysis of HLX and BCL2L11 in L-540 cells after treatment for siRNA-mediated knockdown of HLX (left). Forced expression of HLX in L-428 cells resulted in reduced transcript levels of BCL2L11 (middle). RQ-PCR analysis of L-540 cells treated with STAT3-inhibitor AG490 resulted in increased/induced expression of BCL2L11 (right). (**F**) Reporter-gene assay for the regulation of BCL2L11 expression by HLX. The promoter region of BCL2L11 contains a potential HLX-site (underlined) at −8893 bp (left). RQ-PCR analysis of NIH-3T3 cells transfected with reporter-gene construct and HLX expression construct (right). The activity of the reporter-gene decreased in the presence of HLX, demonstrating direct suppression of BCL2L11 by HLX. (**G**) MTT assay of L-540 cells treated for siRNA-mediated knockdown of HLX showed no significant effect (left). MTT assay of L-540 cells treated for siRNA-mediated knockdown of HLX and concurrently with Etoposide showed strong reduction of viable cell counts, indicating that HLX counteracted Etoposide-induced apoptosis (middle). RQ-PCR analysis of BCL2L11 in L-540 and L-428 cells after treatment with Etoposide demonstrated that Etoposide strongly induced/increased expression of BCL2L11 (right).

Quantification of MSX1 expression in HL cell lines demonstrated nearly undetectable transcript levels in L-540, confirming the profiling data (Figure 5A). To analyze the impact of HLX on MSX1 we performed forced overexpression of HLX in MSX1-expressing L-1236 cells. This approach resulted in decreased MSX1 expression levels, showing that HLX repressed transcription of MSX1. Of note, overexpression of MSX1 in L-540 effected no change in HLX expression levels, discounting reciprocal regulation of these factors (Figure 5A). Treatment of L-540 with TSA suppressed HLX expression (probably via STAT3 translocation, see above), and concordantly induced MSX1 expression, consistent with inhibition of MSX1 transcription by HLX. Likewise, treatment of L-540 with STAT3-inhibitor AG490 simultaneously reduced HLX activity and enhanced MSX1 transcription (Figure 5A).

To analyze the impact of HLX on additional differentiation factors we performed siRNA-mediated knockdown of HLX in L-540. This approach resulted in increased transcription of BCL11A (Figure 5B) and SPIB (Figure 5C). As shown for MSX1, treatment of L-540 with AG490 increased the expression levels of BCL11A and SPIB as well (Figure 5B, 5C). Thus, HLX suppressed the expression of MSX1, BCL11A and SPIB which are all involved in the regulation of B-cell development perturbed in HL [25, 38, 39]. Moreover, profiling data of primary HL and control B-cells indicated reduced expression levels of MSX1, BCL11A and SPIB in HL patient samples, supporting their aberrant downregulation and hence their possible clinical relevance (Supplementary Figure 4, 5). However, the observed difference for MSX1 was not statistically significant.

Finally, we analyzed a potential impact of HLX on the pro-apoptotic factor BCL2L11. Quantification of BCL2L11 transcripts in HL cell lines demonstrated varying levels (Figure 5D). L-540 expressed low/undetectable BCL2L11 levels, confirming the profiling data. Expression analysis of BCL2L11 in primary hematological samples showed enhanced levels in B-cells, indicating cell type specific regulation in the hematopoietic lineages (Figure 5D). SiRNA-mediated knockdown of HLX induced BCL2L11 expression in L-540 cells demonstrating that HLX suppressed BCL2L11 transcription. This regulatory connection was also supported by forced expression of HLX in L-428 and by treatment of L-540 with STAT3-inhibitor AG490 (Figure 5E). Moreover, sequence analysis of the promoter region of BCL2L11 revealed a binding-site for HLX at position −8893 bp. This finding prompted performance of a reporter assay using a corresponding genomic fragment as driver. The combination of the promoter-reporter-construct with an expression-construct encoding HLX indicated direct suppression of BCL2L11 by HLX via this site (Figure 5F). Profiling data of primary HL and control B-cells showed significantly lower expression levels of BCL2L11 in HL patient samples, supporting its aberrant downregulation and clinical relevance (Supplementary Figure 5).

To analyze the functional consequences of the HLX mediated BCL2L11 suppression we quantified viable L-540 cells via MTT assay after HLX knockdown. However, this approach showed no significant difference (Figure 5G). In contrast, simultaneous treatment with Etoposide demonstrated that HLX promoted substantial cell survival under apoptosis-induced conditions (Figure 5G). Etoposide-treatment activated BCL2L11 expression in HL cell lines (Figure 5G), supporting the impact of this gene on apoptosis. Thus, HLX counteracted Etoposide-triggered cell death probably via suppression of pro-apoptotic factor BCL2L11. Taken together, this target gene analysis revealed that HLX suppressed B-cell differentiation factors MSX1, SPIB and BCL11A and pro-apoptotic factor BCL2L11. Consistently, disturbed differentiation and inhibited apoptosis represent fundamental mechanisms for the pathology of HL.

## DISCUSSION

**Figure 6:**
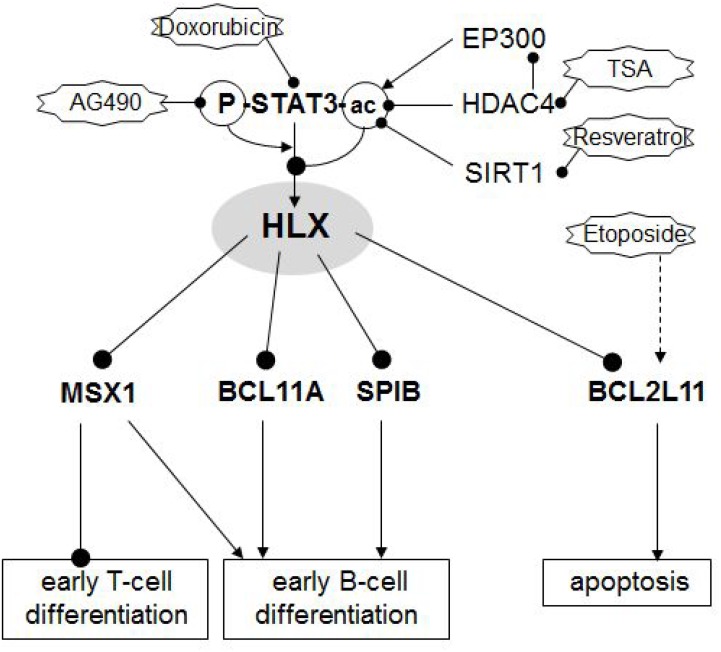
Gene regulatory network of HLX Schematic summary of the results obtained in this study. NKL homeobox gene HLX is located at a central position of a pathological gene regulatory network. HLX is activated by phosphorylated and deacetylated STAT3. Acetylation of STAT3 protein mediates nuclear export of the protein and subsequent loss of its gene activating potential. STAT3-acetylation may occur by EP300 (downregulated in HL), deacetylation by HDAC4 (overexpressed in HL) and SIRT1. Target genes regulated by HLX are differentiation factors MSX1, BCL11A and SPIB and pro-apoptotic factor BCL2L11/BIM.

The results of our study are summarized schematically in Figure 6. Screening of profiling data from patients revealed aberrant activity of NKL homeobox gene HLX in a subset of cHL. Experimental analysis of HLX expressing HL cell line L-540 indicated that STAT3 activated HLX transcription and the subcellular localization of STAT3 was regulated by acetylation. HLX in turn repressed particular B-cell differentiation factors and Etoposide-induced apoptosis via BCL2L11.

Homeobox genes of the NKL subclass encode basic developmental TFs showing tissue specific expression patterns [40]. Accordingly, pharyngeal and hematopoietic NKL-codes have been described [25, 32]. Many NKL homeobox genes are aberrantly overexpressed in T-ALL, representing an important class of oncogenes in this lymphoid malignancy [24, 25, 28]. Deregulated NKL homeobox genes have been described in B-cell malignancies as well including MSX1 in mantle cell lymphoma, NKX2-1 in DLBCL, and NKX2-3 in marginal zone B-cell lymphoma [41–43]. Here, we identified aberrant expression of HLX in HL, of HLX and NKX2-3 in DLBCL, and of NKX6-3 in BL, extending the list of deregulated NKL homeobox genes in B-cell malignancies. HLX is physiologically expressed in early hematopoiesis including HSCs and lymphoid progenitors [25, 33]. Forced expression of HLX in the lymphoid lineage of mice resulted in disturbed differentiation of B- and T-cells, showing that HLX inhibits the development of lymphocytes [44]. Aberrant expression of HLX has been found in 87% of acute myeloid leukemia (AML) patients and is correlated with inferior overall survival [45]. This high incidence might indicate a predisposition of the myeloid lineage for transformation by this oncogene. In AML HLX generates a differentiation stop as well, recapitulating its basic function conducted in progenitor and stem cells [45]. Of note, NKL homeobox genes HLX, NKX2-1, NKX2-3, NKX6-3 and MSX1 are deregulated in B-cell lymphoma and show aberrant activity in T-ALL as well which might indicate similar oncogenic functions in both lineages of lymphoid malignancies [25]. In T-ALL a correlation has been described between aberrant NKL homeobox gene expression and stage-specific differentiation stop [23]. Therefore, we conclude that a similar relation might also be valid for B-cell tumors including HL.

HLX itself in addition to three target genes identified in this study (MSX1, SPIB and BCL11A) are implicated in B-cell differentiation. NKL homeobox gene MSX1 is physiologically expressed in CLPs, elevated in B-cell progenitors, downregulated during the maturation of B- and T-cells, and constantly active in NK-cells [25, 26, 46]. Deregulated expression of MSX1 has been described previously in HL, however, the gene acts in this malignancy either as tumor suppressor (TS) or oncogene, a duality which might depend on interacting cofactors [47]. Furthermore, MSX1 has been shown in HL as a target gene of two additional oncogenic non-NKL homeodomain factors, namely SIX1 and OTX2 [48, 49]. Interestingly, MSX1, SIX1 and OTX2 represent embryonic regulators of the neural plate border region and its descendants, indicating aberrant reactivation of a particular developmental gene network in HL by these factors [50]. Two additional deregulated B-cell factors identified in this study were SPIB and BCL11A. SPIB regulates B-cell differentiation and acts as an oncogene in ABC-DLBCL [39, 51]. Here, we show that aberrant activity of HLX in HL inhibits the expression of SPIB, suggesting that SPIB operates as TS in HL. Consistently, SPIB is a target gene of PAX5 in early B-cell differentiation [52]. Thus, aberrant downregulation of PAX5 in HL may contribute to the observed reduction of SPIB expression as well, supporting the TS status for SPIB in this type of B-cell malignancy. BCL11A is expressed in HSCs, CLPs and B/T-cell progenitors [53]. Consequently, BCL11A impacts differentiation processes in B-cells [54]. In HL coamplification of BCL11A has been detected together with its neighbor REL, but BCL11A has been excluded as target gene of this activating genomic aberration [12].

Oncogenic NKL expression correlated with aberrant survival pathways in T-ALL [25]. Consistent with this observation HLX was found to enhance survival via suppression of the pro-apoptotic factor BCL2L11 in HL. BCL2L11 deletion and TS gene activity has been described in B-NHLs [55]. BCL2L11 is responsible for Etoposide-mediated apoptosis and states a risk gene in NHL [56, 57]. Deregulation of pro- and anti-apoptotic BCL2-like proteins constitutes an important pathological mechanism in HL. Accordingly, the observed correlation of pro-apoptotic BCL2L11 and anti-apoptotic MCL1 indicates that MCL1 mechanistically counteracts BCL2L11 [58]. Thus, BCL2L11 represents a major factor mediating apoptosis in HL. Its suppression critically contributes to the aberrant survival of HRS cells.

The JAK/STAT pathway in general and STAT3 in particular play essential pathological roles in multiple cancers including HL [10, 11, 59]. Correspondingly, amplification and overexpression of STAT3-activator JAK2 and enhanced levels of phosphorylated STAT3 have been described in HL cell lines [9, 10]. Furthermore, several types of aberrations affecting STAT-inhibitors have been found in HL which enhance the signalling of this pathway [60, 61]. Finally, STAT3 is a useful classifier to differentiate the related hematopoietic malignancies HL and anaplastic large cell lymphoma [35]. Thus, STAT3 represents a key factor for the pathogenesis of HL. Target gene analysis of STAT3 in the ABC-type of DLBCL showed that HLX is directly activated by STAT3, indicating a general regulatory connection in lymphocytes [62]. The same study revealed for STAT3 suppression of several genes involved in apoptosis as well, indicating that both HLX and STAT3 mediate survival in HL.

The subcellular/nuclear localization of STAT3 is regulated via acetylation at particular sites. STAT3 acetylation at position K87 mediates its translocation into mitochondria in fibroblasts [63], while acetylation at position K685 mediates nuclear translocation, dimerization and transcriptional activation in PC-3 cells [36, 64]. In contrast, our data showed acetylation-mediated cytoplasmic localization, indicating that cell type specific characteristics are responsible for the particular consequences of STAT3 acetylation. The study of underlying mechanisms performing acetylation-mediated subcellular localizations might resolve these conflicting results. Deacetylation of non-histone proteins including STAT3 is conducted by both HDACs and SIRT1 [37, 65]. Our data highlight overexpressed HDAC3 as possible factor mediating STAT3 deacetylation in HL patients and cell line L-540. While STAT3 activation has been reported in many HL cell lines, L-540 exhibited the most intense signal for phosphorylated STAT3 [10]. Enhanced activation together with nuclear concentration of STAT3 protein may represent major requirements for HLX expression in HL. Thus, aberrant expression of HLX in HL depends on powerful STAT3-signalling and elevated deacetylase activity. However, our data can not exclude an impact of additional factors regulating HLX expression.

In conclusion, our study highlights HLX deregulation in HL subsets, extending the oncogenic potential of NKL homeobox genes in B-cell lymphoma. The consequences of aberrant HLX expression include deregulated B-cell differentiation and reduced apoptosis, resembling oncogenic functions of deregulated NKL subclass members in T-ALL. The data suggest that HLX positive cHL cases may have worse prognosis and require alternative treatment protocols-supplementing HDAC-inhibitors may represent a promising option for their therapy.

## MATERIALS AND METHODS

### Expression profiling and bioinformatics

Public expression profiling datasets of lymphoma/leukemia patients were obtained from Gene Expression Omnibus (GEO; www.ncbi.nlm.nih.gov/gds): GSE12453 (containing samples of HL, B-NHL and normal B-cells) [66], GSE39134 (29 cHL samples) [67], and GSE25990 (GC biopsies, HL cell lines) [68]. These gene expression microarray profiling data were generated using the HG U133 Plus 2.0 gene chip (Affymetrix, High Wycombe, UK). Expression values were given as barplots obtained directly from the website or as boxplots using R-packages (http://www.bioconductor.org/). Statistical significance was calculated by the Students *T*-Test and obtained *P*-values are indicated.

Expression profiling datasets of HL cell lines (HG U133 Plus 2.0 gene chip, Affymetrix) were generated by Prof. Andreas Rosenwald (Institute of Pathology, University of Würzburg, Germany) and by Dr. Robert Geffers (Genome Analytics, Helmholtz Centre for Infection Research, Braunschweig, Germany). After RMA-background correction and quantile normalization of the spot intensities, the profiling data were expressed as ratios of the sample mean and subsequently log2 transformed. Data processing was performed via R/Bioconductor using limma and affy packages. The profiling data of the cell lines have been published recently [51]. To parse biological function of shortlisted genes, gene-annotation enrichment analysis was performed using DAVID bioinformatics resources [69].

### Cell lines and treatments

HL-cell lines HDLM-2, KM-H2, L-428, L-540, L-1236, SUP-HD1, U-HO1 and AML cell line THP-1 are held by the DSMZ (Braunschweig, Germany) and cultivated as described [70]. Cell stimulations were performed for 16 h by treatment with 20 ng/ml recombinant human proteins IGF1, IFNG, IL2, IL6, IL7, IL12, IL21, IL22 or IL23 (R&D Systems, Wiesbaden, Germany), with inhibitory antibodies directed against IGF1, IFNGR1, IFNGR2, IL7, IL22 (R&D Systems), with 10 or 100 nM 5-Azacytidine (AZA, Sigma, Taufkirchen, Germany), 5 μM DZNep (Sigma), 10 μg/ml Trichostatin A (TSA, Sigma), 50 μM Resveratrol (Sigma), 0.5 μM ICBP112 (Sigma), 1 μM Epinephrine (Sigma), 1 μM Propranolol (Sigma), Doxorubicine (Sigma), AG490 (Sigma), and Etoposide (Sigma). Gene specific siRNA oligonucleotides and AllStars negative Control siRNA (siControl) were obtained from Qiagen (Hilden, Germany). Expression constructs for HLX, MSX1, STAT3, and GFP-tagged STAT3 were obtained from Origene (Wiesbaden, Germany). SiRNAs (80 pmol) and expression constructs/vector controls (2 μg) were transfected into 1 × 10^6^ cells by electroporation using the EPI-2500 impulse generator (Fischer, Heidelberg, Germany) at 350 V for 10 ms. Transfected cells were harvested after 20 h cultivation.

### Genomic and chromosomal analyses

For genomic profiling genomic DNA of the HL-cell lines was prepared by the Qiagen Gentra Puregene Kit (Qiagen). Labelling, hybridization and scanning were performed at the Genome Analytics Facility, Helmholtz Centre for Infection Research (Braunschweig, Germany), according to the manufacturer´s protocols (Affymetrix). Data were interpreted using the Chromosome Analysis Suite software version 2.0.1.2 (Affymetrix).

Chromosomal analysis by FISH was performed as described previously [71]. BAC clones were obtained from BacPac Resources, Childrenʼs Hospital Oakland Research Institute (CA, USA) to analyze HLX (RP11-28I6, RP11-780G17, RP11-121E10). Insert DNA was harvested using the Big BAC DNA Kit (Princeton Separations, Adelphia, NJ, USA) and directly labelled by nick translation with dUTP-fluors (Dyomics, Jena, Germany). Whole chromosome 1 painting probe was obtained from Applied Spectral Imaging (Neckarhausen, Germany). Fluorescent images were captured and analyzed with an Axio-Imager microscope (Zeiss, Göttingen, Germany) configured to a dual Spectral Imaging FISH system (Applied Spectral Imaging).

### Polymerase chain-reaction (PCR) analyses

Total RNA was extracted from cell line samples using TRIzol reagent (Invitrogen, Darmstadt, Germany). Primary human total RNA used in this study was commercially obtained-isolated from peripheral blood mononuclear cells (PBC), thymus, lymph node (LN), spleen, and bone marrow (BM) from Biochain/BioCat (Heidelberg, Germany), and RNA from peripheral CD19-positive B-cells and CD3-positive T-cells from Miltenyi Biotec (Bergisch Gladbach, Germany). RNA from CD34-positive hematopoietic stem cells (HSCs) was obtained from Prof. Michaela Scherr as described previously [25]. cDNA was synthesized from 5 μg RNA by random priming using Superscript II (Invitrogen). Real-time quantitative (RQ)-PCR analysis was performed with the 7500 Real-time System, using commercial buffer and primer sets (Thermo Fisher Scientific, Darmstadt, Germany). Quantification of MSX1 was performed as described previously [72]. For normalization of expression levels we analyzed the transcript of TATA box binding protein (TBP). We used the ddCT-method and the obtained values are indicated in relation to one sample which was set to 1.

Quantitative analyses were performed in triplicate. Standard deviations are presented in the figures as error bars. The statistical significance was assessed by *T*-Test and the calculated *p*-values indicated by asterisks (^*^p < 0.05, ^**^p < 0.01, ^***^p < 0.001, n.s. not significant).

### Sequence analysis

cDNA used for sequencing STAT3 of cell line L-540 was generated as described above. Amplification of the SH2-domain of STAT3 which contains a described acetylation-site was performed by PCR, using the thermocycler TGradient (Biometra, Göttingen, Germany) and the following oligonucleotides: STAT3-for 5ʹ-CATGGGCTTTATCAGTAAGGAGCGG-3ʹ, and STAT3-rev 5ʹ-CACAGATAAACTTGGTCTTCAGGTA TGG-3ʹ (Eurofins MWG, Ebersbach, Germany). The PCR product (385 bp) was cloned into pGEM-T Easy (Promega, Mannheim, Germany) and sequenced at Eurofins MWG. The obtained sequence data were analyzed by BLAST (blast.ncbi.nlm.nih.gov).

### Protein analyses

Western blots were generated by the semi-dry method. Protein lysates from cell lines were prepared using SIGMAFast protease inhibitor cocktail (Sigma). Proteins were transferred onto nitrocellulose membranes (Bio-Rad, München, Germany) and blocked with 5% dry milk powder dissolved in phosphate-buffered-saline buffer (PBS). The following antibodies were used: alpha-Tubulin (Sigma), HLX (Novus Biologicals, Abingdon, UK), STAT3 and phospho-STAT3 (Cell Signalling, Leiden, Netherlands). For loading control blots were reversibly stained with Poinceau (Sigma) and detection of alpha-Tubulin (TUBA) was performed thereafter. Secondary antibodies were linked to peroxidase for detection by Western-Lightning-ECL (Perkin Elmer, Waltham, MA, USA). Documentation was performed using the digital system ChemoStar Imager (INTAS, Göttingen, Germany).

Immuno-cytology was performed as follows: cells were spun onto slides and subsequently air-dried and fixed with methanol/acetic acid for 90 s. The antibodies were diluted 1:20 in PBS containing 5% BSA and incubated for 30 min. Washing was performed 3 times with PBS. Preparations were incubated with secondary antibody (diluted 1:100) for 20 min. After final washing the cells were mounted in Vectashield (Vector Laboratories, Burlingame, CA), containing DAPI for nuclear staining. Documentation of subcellular protein localization was performed using fluorescence microscope Axiovert 40 CFL (Zeiss) and software VisiView version 1.6.9 (Visitron Systems, Puchheim, Germany).

### Reporter gene assay

For creation of reporter gene constructs we combined a reporter with a regulatory genomic fragment derived from the promoter regions of HLX and BCL2L11, containing potential binding sites for STAT3 and HLX, respectively [73]. We cloned the genomic PCR products of the corresponding promoter regions (regulator) and of the HOXA9 gene, comprising exon1-intron1-exon2 (reporter), into the *Hind*III/*Bam*HI and *Eco*RI sites, respectively, of the expression vector pcDNA3 downstream of the CMV enhancer. The oligonucleotides used for the amplification of the regulators were obtained from Eurofins MWG. Their sequences were as follows: HLX-for 5ʹ-GGAAGCTTGTGCCGCTCTCCCGGGTTTCG-3ʹ, HLX-rev 5ʹ-TTGGATCCAGGAGTCCGTGTCCTCGGCAAAGC-3ʹ. BCL2L11-for 5ʹ- TAAAGCTTGGAAGTGGCAATGTCAGAGTTC −3ʹ, BCL2L11-rev 5ʹ-CAGGATCCAGGTCTATTAAATAATCAGGACTCCAGC-3ʹ. Intro-duced restriction sites used for cloning are underlined. The obtained PCR products had a size of 331 and 350 bp, respectively. Constructs were validated by sequence analysis (Eurofins MWG). Transfections of plasmid-DNA into NIH-3T3 cells (DSMZ ACC 59) were performed using SuperFect Transfection Reagent (Qiagen). Commercial HOXA9 and TBP assays were used for RQ-PCR to quantify the spliced reporter-transcript, corresponding to the regulator activity. A cotransfected commercial luciferase construct served as transfection control and was quantified by the Luciferase Assay System (Promega, Mannheim, Germany) using the luminometer Lumat LB9501 (Berthold Technologies, Bad Wildbad, Germany).

### MTT assay

HL cell lines were transfected as indicated and and treated for 20 h with 10 μg/ml Etoposide (Sigma) which has been dissolved in dimethylsulfoxide, and subsequently prepared for standardized MTT (3-(4, 5-dimethylthiazol-2-yl)-2, 5-diphenyltetrazolium bromide; obtained from Sigma) assays. The measurement was performed twice in triplicates. The absorbance was determined at 540 nm and at 620 nm as background control using ELISA reader Multiskan EX (Thermo Electron, Vantaa, Finland).

## SUPPLEMENTARY MATERIALS FIGURES AND TABLES






