# Supplementary material for: Aberrant expression of NKL homeobox gene HLX in Hodgkin lymphoma
Source: Oncotarget. 2018 Feb 16;9(18):14338–53. doi: 10.18632/oncotarget.24512 (PMC5865674; doi:10.18632/oncotarget.24512)
Supplement: Supplementary file 1 [file oncotarget-09-14338-s001.pdf]

Aberrant expression of NKL homeobox gene HLX in Hodgkin lymphoma

SUPPLEMENTARY MATERIALS

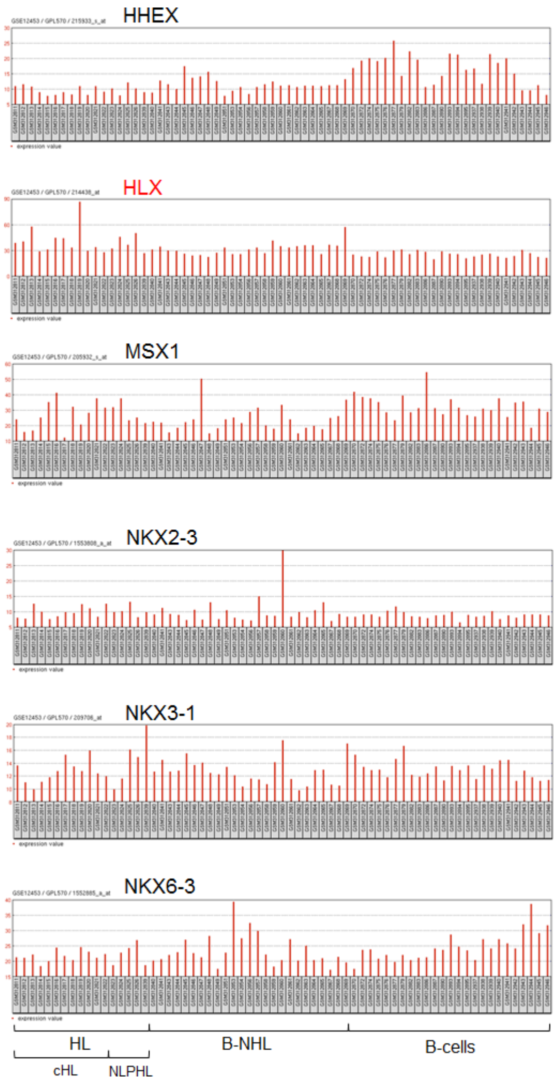

Supplementary Figure 1: NKL homeobox gene profiling in B-cell lymphoma patients. Public gene expression profiling dataset GSE12453 contains in addition to 17 microdissected HL cases (12 cHL and 5 NLPHL), 25 B-NHL cases (4 TCRBCL, 5 FL, 5 BL and 11 DLBCL) and 25 normal B-cell samples representing different developmental stages obtained from peripheral blood and tonsils [67]. The transcript levels for six NKL homeobox genes were analyzed in this dataset, demonstrating aberrantly elevated expression of HLX in HL patient samples.

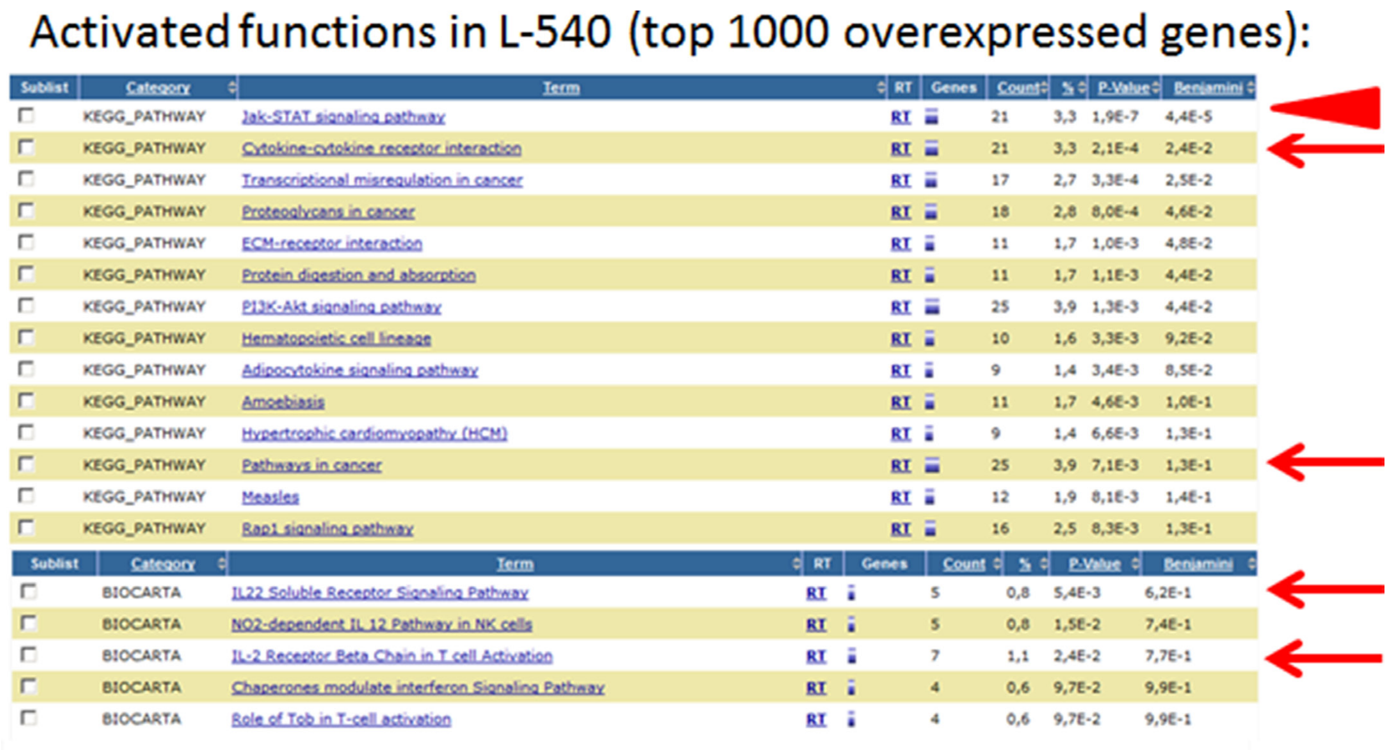

Activated functions in L-540 (top 1000 overexpressed genes):

Sublist	Category	Term	RT	Genes	Count	%	P-Value	Benjamini
	KEGG_PATHWAY	Jak-STAT signaling pathway	RT		21	3,3	1,9E-7	4,4E-5
	KEGG_PATHWAY	Cytokine-cytokine receptor interaction	RT		21	3,3	2,1E-4	2,4E-2
	KEGG_PATHWAY	Transcriptional misregulation in cancer	RT		17	2,7	3,3E-4	2,5E-2
	KEGG_PATHWAY	Proteoglycans in cancer	RT		18	2,8	8,0E-4	4,6E-2
	KEGG_PATHWAY	ECM-receptor interaction	RT		11	1,7	1,0E-3	4,8E-2
	KEGG_PATHWAY	Protein digestion and absorption	RT		11	1,7	1,1E-3	4,4E-2
	KEGG_PATHWAY	PI3K-Akt signaling pathway	RT		25	3,9	1,3E-3	4,4E-2
	KEGG_PATHWAY	Hematopoietic cell lineage	RT		10	1,6	3,3E-3	9,2E-2
	KEGG_PATHWAY	Adipocytokine signaling pathway	RT		9	1,4	3,4E-3	8,5E-2
	KEGG_PATHWAY	Amoebiasis	RT		11	1,7	4,6E-3	1,0E-1
	KEGG_PATHWAY	Hypertrophic cardiomyopathy (HCM)	RT		9	1,4	6,6E-3	1,3E-1
	KEGG_PATHWAY	Pathways in cancer	RT		25	3,9	7,1E-3	1,3E-1
	KEGG_PATHWAY	Measles	RT		12	1,9	8,1E-3	1,4E-1
	KEGG_PATHWAY	Rap1 signaling pathway	RT		16	2,5	8,3E-3	1,3E-1

Sublist	Category	Term	RT	Genes	Count	%	P-Value	Benjamini
	BIOCARTA	IL22 Soluble Receptor Signaling Pathway	RT		5	0,8	5,4E-3	6,2E-1
	BIOCARTA	NO2-dependent IL 12 Pathway in NK cells	RT		5	0,8	1,5E-2	7,4E-1
	BIOCARTA	IL-2 Receptor Beta Chain in T cell Activation	RT		7	1,1	2,4E-2	7,7E-1
	BIOCARTA	Chaperones modulate interferon Signaling Pathway	RT		4	0,6	9,7E-2	9,9E-1
	BIOCARTA	Role of Tob in T-cell activation	RT		4	0,6	9,7E-2	9,9E-1

Supplementary Figure 2: Gene-annotation enrichment analysis data (DAVID). The top 1000 overexpressed genes in L-540 as compared to five control HL cell lines were analyzed using the online tool DAVID provided by the National Institute of Health. The results show significantly high representation of KEGG-pathway term JAK-STAT signaling pathway (arrow head) in addition to further four terms of other signaling pathways from KEGG-pathway and BIOCARTA (arrows).

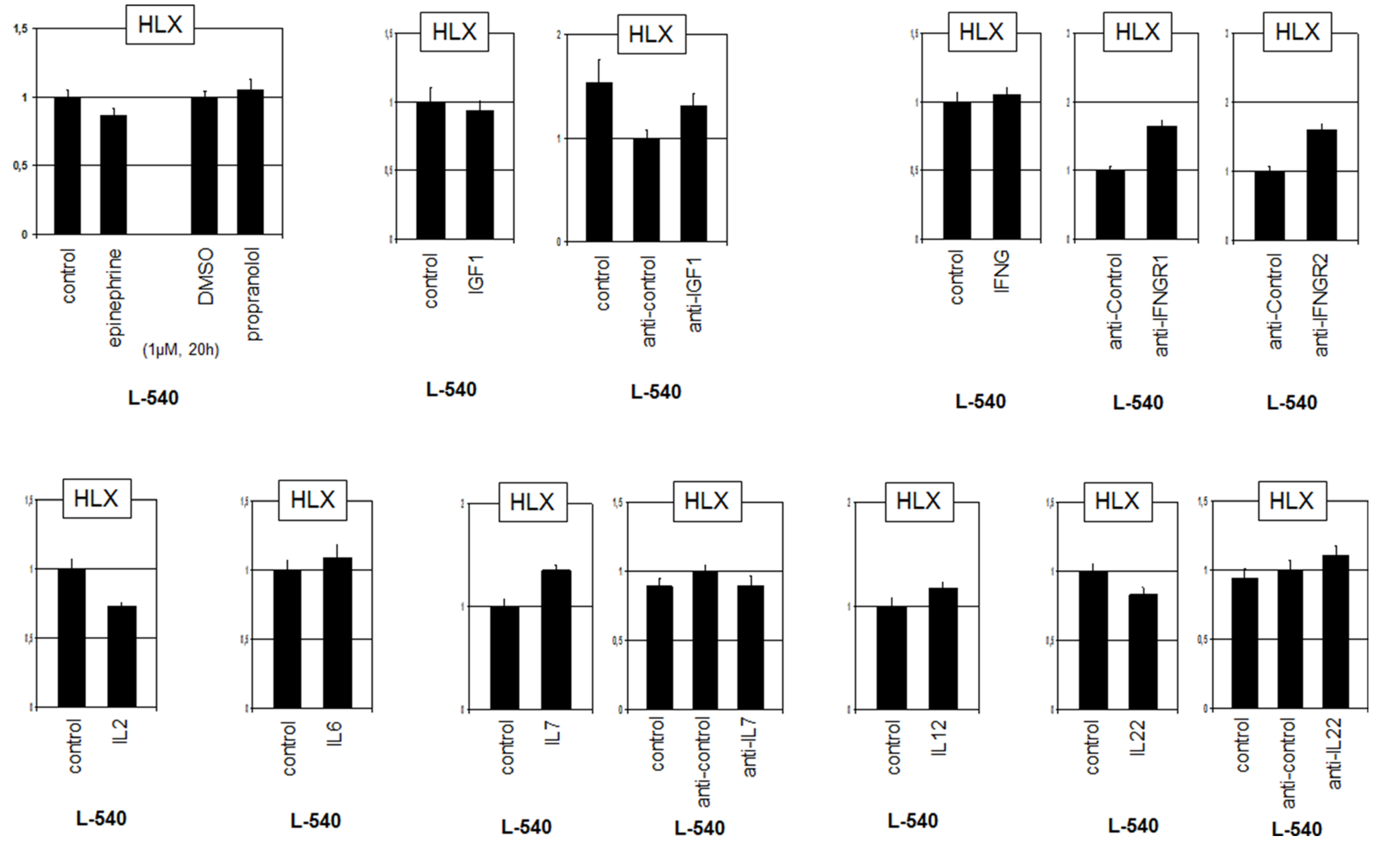

Supplementary Figure 3: Treatments of HL cell line. HL cell line L-540 was treated with two drugs activating/inhibiting adrenoreceptor signaling, with different recombinant receptor-ligands, and with inhibitory antibodies directed against particular ligands or receptors. The expression of HLX was subsequently quantified by RQ-PCR. However, no significant alteration was detected which would have indicated HLX activation.

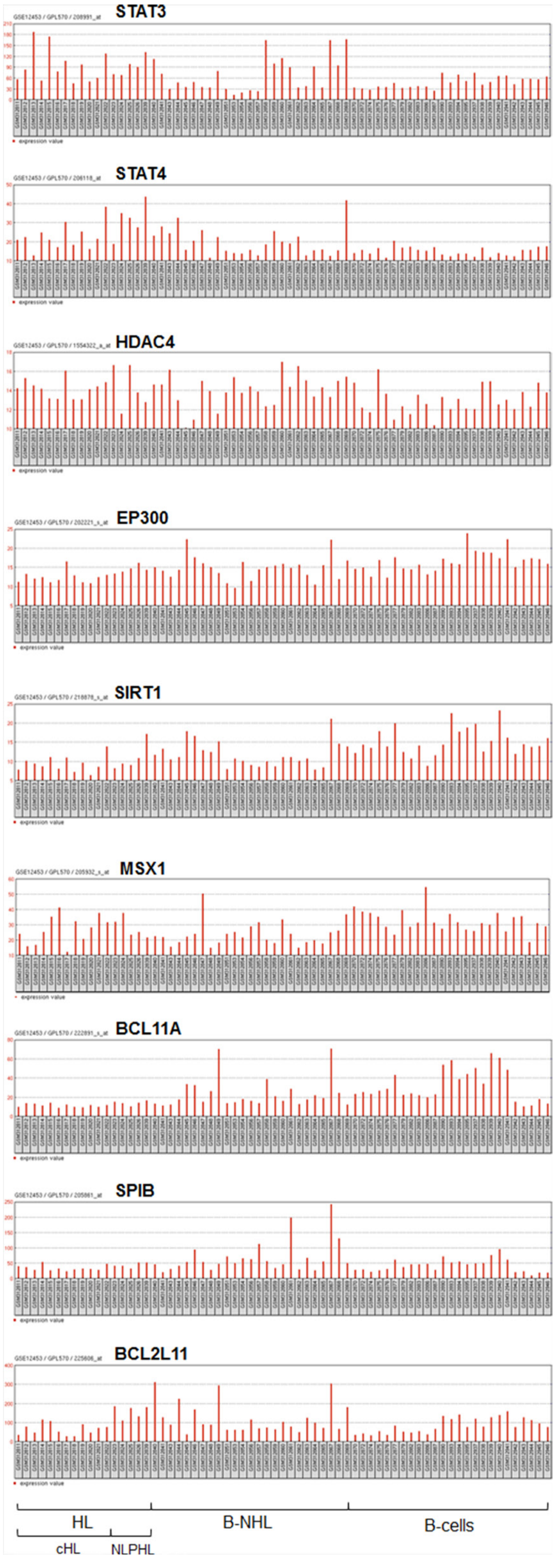

Supplementary Figure 4: Gene expression profiling data in HL patients. Public gene expression profiling dataset GSE12453 contains in addition to 17 microdissected HL cases (12 cHL and 5 NLPHL), 25 B-NHL cases (4 TCRBCL, 5 FL, 5 BL and 11 DLBCL) and 25 normal B-cell samples representing different developmental stages obtained from peripheral blood and tonsils [67]. The transcript levels for potential upstream regulators and downstream target genes of HLX were analyzed in this dataset.

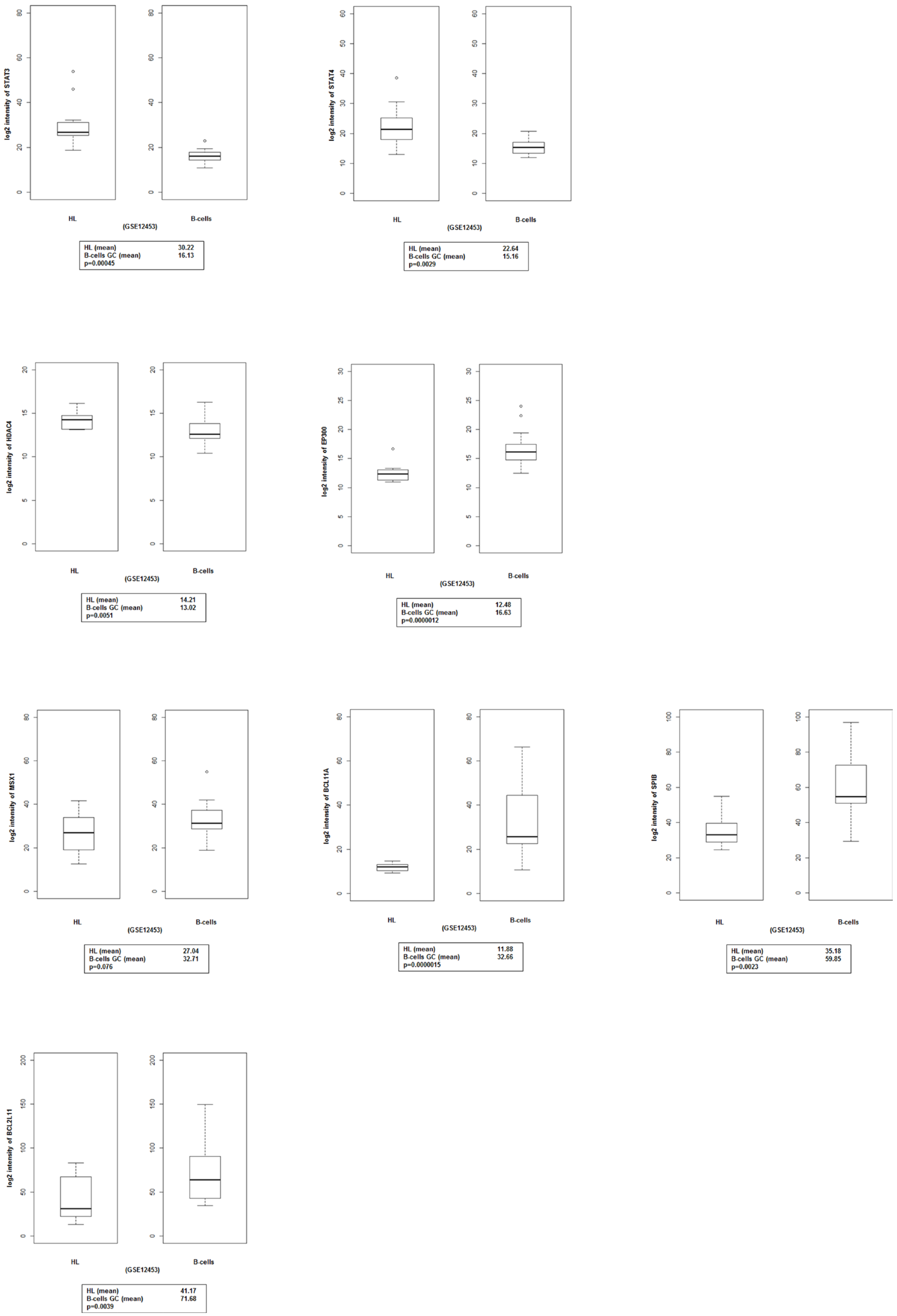

Supplementary Figure 5: Gene expression statistics. Expression data of 17 HL patient samples and of 25 B-cell controls obtained from public gene expression profiling dataset GSE12453 were analyzed for potential upstream regulators and downstream target genes of HLX. The expression levels are indicated as boxplot and the statistical significance of expression level differences were analyzed by Students *T*-test – the calculated *p*-values are indicated.

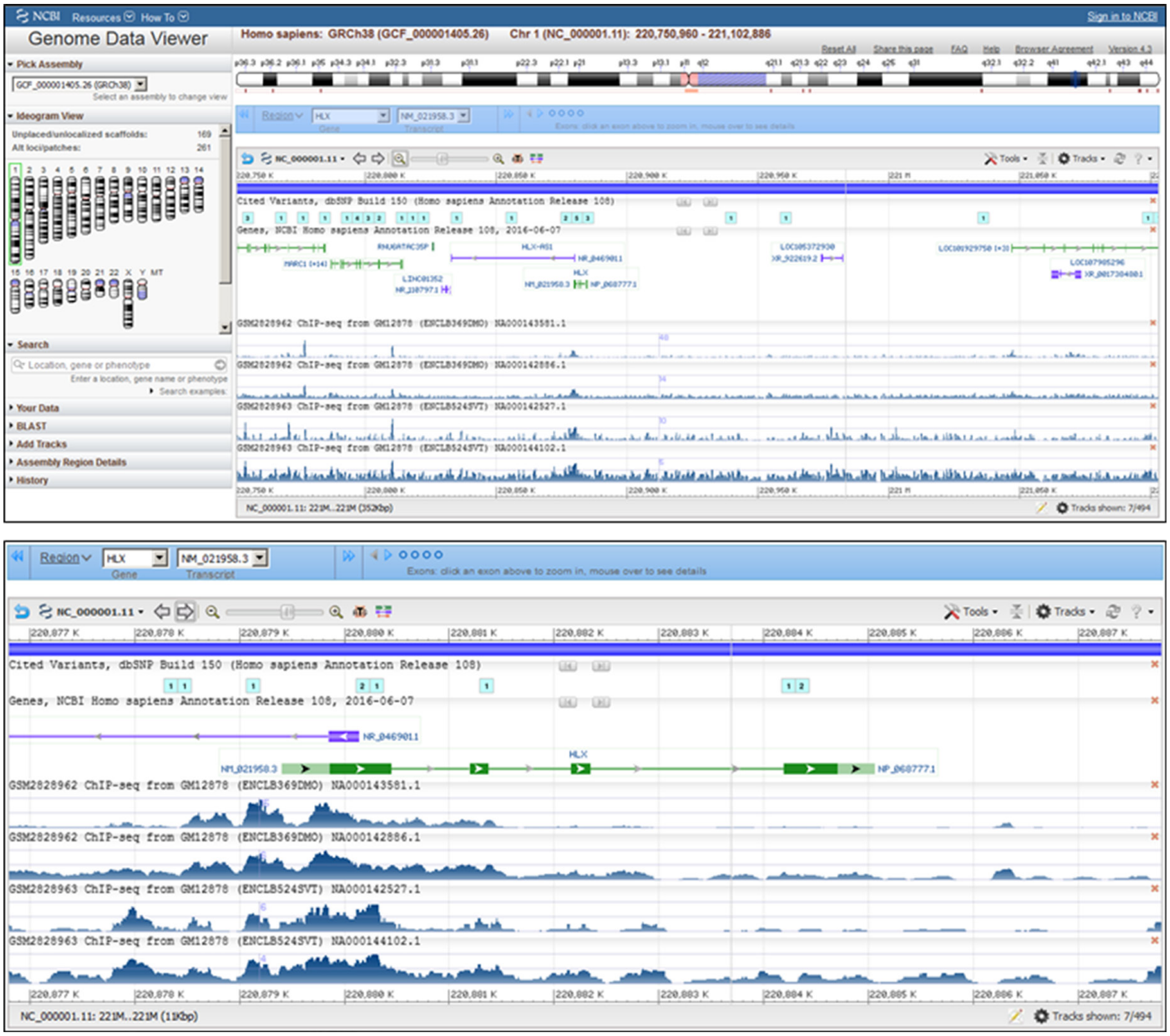

Supplementary Figure 6: STAT3 ChIP-seq data. Public Chip-seq dataset GM12878 obtained from the ENCODE project was analyzed for STAT3 binding, showing interaction at the HLX gene (above). The focused view (below) shows STAT3 binding in the promoter region of HLX.

Supplementary Table 1: Comparative expression profiling. See Supplementary_Table_1

Supplementary Table 2: Comparative expression profiling. See Supplementary_Table_2
